# Forecasting Future Asthma Hospital Encounters of Patients With Asthma in an Academic Health Care System: Predictive Model Development and Secondary Analysis Study

**DOI:** 10.2196/22796

**Published:** 2021-04-16

**Authors:** Yao Tong, Amanda I Messinger, Adam B Wilcox, Sean D Mooney, Giana H Davidson, Pradeep Suri, Gang Luo

**Affiliations:** 1 Department of Biomedical Informatics and Medical Education University of Washington Seattle, WA United States; 2 The Breathing Institute Department of Pediatrics University of Colorado School of Medicine, Children’s Hospital Colorado Aurora, CO United States; 3 Department of Surgery University of Washington Seattle, WA United States; 4 Department of Health Services University of Washington Seattle, WA United States; 5 Seattle Epidemiologic Research and Information Center & Division of Rehabilitation Care Services VA Puget Sound Health Care System Seattle, WA United States; 6 Clinical Learning, Evidence, and Research (CLEAR) Center University of Washington Seattle, WA United States; 7 Department of Rehabilitation Medicine University of Washington Seattle, WA United States

**Keywords:** asthma, forecasting, machine learning, patient care management, risk factors

## Abstract

**Background:**

Asthma affects a large proportion of the population and leads to many hospital encounters involving both hospitalizations and emergency department visits every year. To lower the number of such encounters, many health care systems and health plans deploy predictive models to prospectively identify patients at high risk and offer them care management services for preventive care. However, the previous models do not have sufficient accuracy for serving this purpose well. Embracing the modeling strategy of examining many candidate features, we built a new machine learning model to forecast future asthma hospital encounters of patients with asthma at Intermountain Healthcare, a nonacademic health care system. This model is more accurate than the previously published models. However, it is unclear how well our modeling strategy generalizes to academic health care systems, whose patient composition differs from that of Intermountain Healthcare.

**Objective:**

This study aims to evaluate the generalizability of our modeling strategy to the University of Washington Medicine (UWM), an academic health care system.

**Methods:**

All adult patients with asthma who visited UWM facilities between 2011 and 2018 served as the patient cohort. We considered 234 candidate features. Through a secondary analysis of 82,888 UWM data instances from 2011 to 2018, we built a machine learning model to forecast asthma hospital encounters of patients with asthma in the subsequent 12 months.

**Results:**

Our UWM model yielded an area under the receiver operating characteristic curve (AUC) of 0.902. When placing the cutoff point for making binary classification at the top 10% (1464/14,644) of patients with asthma with the largest forecasted risk, our UWM model yielded an accuracy of 90.6% (13,268/14,644), a sensitivity of 70.2% (153/218), and a specificity of 90.91% (13,115/14,426).

**Conclusions:**

Our modeling strategy showed excellent generalizability to the UWM, leading to a model with an AUC that is higher than all of the AUCs previously reported in the literature for forecasting asthma hospital encounters. After further optimization, our model could be used to facilitate the efficient and effective allocation of asthma care management resources to improve outcomes.

**International Registered Report Identifier (IRRID):**

RR2-10.2196/resprot.5039

## Introduction

### Background

In the United States, 7.7% of people have asthma, which causes 188,968 hospitalizations, 1,776,851 emergency department (ED) visits, and 3441 deaths annually [1]. To reduce asthma hospital encounters covering both hospitalizations and ED visits, many health care systems and health plans deploy predictive models to prospectively find patients at high risk and offer them care management services for preventive care. The University of Washington Medicine (UWM), Intermountain Healthcare, and Kaiser Permanente Northern California [2] are 3 examples of such health care systems. Examples of such health plans include those in 9 of the 12 metropolitan communities [3]. Once a patient is deemed to be at high risk and enrolled in a care management program, a care manager will regularly assess the patient’s asthma control, adjust the patient’s asthma medications if necessary, and help the patient make appointments for health and related services. Using effective care management, as many as 40% of future hospital encounters by patients with asthma can be avoided [4-7].

Owing to its limited service capacity, a care management program normally enrolls at most 3% of patients with a particular condition [8]. To maximize the benefits of this resource-intensive program, it is crucial for the program to only enroll the patients who are at the highest risk. After all, the deployed predictive model’s accuracy (or lack thereof) places an upper bound on the program’s effectiveness. Several other research groups have built multiple models for forecasting future asthma hospital encounters of patients with asthma. Every model examined only a few features [2,9-22]. Overlooking some important features in the model degrades model accuracy, making the model miss at least half of the patients who will experience future asthma hospital encounters and incorrectly forecast future asthma hospital encounters for many other patients with asthma. These errors result in impaired patient outcomes and wasted health care spending [23]. In nonmedical fields, people frequently adopt the modeling strategy of examining many candidate features to enhance the accuracy of machine learning models [24-27]. Embracing this modeling strategy for medical data, we built a new machine learning model to forecast future asthma hospital encounters of patients with asthma at Intermountain Healthcare, a nonacademic health care system [23]. Our Intermountain Healthcare model raised the area under the receiver operating characteristic curve (AUC) to 0.859, which is higher than that of every previously published model by 0.049 or more. Although this progress is encouraging, it is unclear how well our modeling strategy generalizes to academic health care systems, which normally care for more complex and sicker patients than nonacademic health care systems [28].

### Objective

This study evaluates the generalizability of our modeling strategy to the UWM, an academic health care system. Similar to the Intermountain Healthcare model [23], our UWM model uses clinical and administrative data to forecast future asthma hospital encounters of patients with asthma covering both hospitalizations and ED visits. There are 2 possible values of the categorical dependent variable: whether the patient with asthma will experience asthma hospital encounters in the subsequent 12 months. This paper reports on the development and evaluation of the UWM model.

### Our Contributions

This study makes the following 3 innovative contributions:

1. We conducted the first evaluation of the generalizability of our modeling strategy to an academic health care system.
2. We evaluated the predictive power of 71 new features, which were not used in our previous study [23], for forecasting asthma hospital encounters.
3. We evaluated the generalizability of our Intermountain Healthcare model to the UWM and the generalizability of our UWM model to Intermountain Healthcare. To the best of our knowledge, this is the first study to evaluate model generalizability in both directions. Previously, model generalizability was evaluated solely in one direction by assessing the performance of a model built using one site’s data on another site’s data [17].

## Methods

### Study Design and Ethics Approval

The institutional review boards of the UWM and Intermountain Healthcare approved this secondary analysis study on clinical and administrative data.

### Patient Cohort

The UWM is the largest academic health care system in Washington State. Its enterprise data warehouse contains clinical and administrative data from 3 hospitals and 12 clinics for adults. Our patient cohort covered adult patients with asthma (age ≥18 years) who visited any of these UWM facilities between 2011 and 2018. We defined a patient as having asthma in a specific year if the encounter billing database contained at least one asthma diagnosis code (International Classification of Diseases, Ninth Revision [ICD-9]: 493.0x, 493.1x, 493.8x, 493.9x; International Classification of Diseases, Tenth Revision [ICD-10]: J45.x) record of the patient in that year [10,29,30]. As the sole exclusion criterion, we eliminated patients who passed away in that year.

### Prediction Target (Dependent Variable)

**Figure 1:**
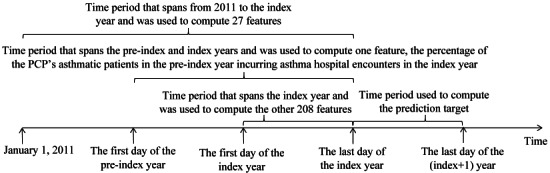
The time periods used to compute the features and prediction target for an index year and patient pair. PCP: primary care provider.

The prediction target was from our previous study [23]. We defined an asthma hospital encounter as a hospitalization or an ED visit with asthma as its principal diagnosis (ICD-9: 493.0x, 493.1x, 493.8x, 493.9x; ICD-10: J45.x). As Figure 1 shows, for each patient deemed to have asthma in a specific year, we used any asthma hospital encounter at UWM in the subsequent 12 months, that is, the 12 months after the end of this year, as the outcome of interest. We adopted the patient’s data by the end of this year to forecast the patient’s outcome in the subsequent 12 months.

### Data Set

The UWM enterprise data warehouse supplied a structured data set that contained clinical and administrative data on our patient cohort’s encounters at the 3 UWM hospitals and 12 UWM clinics between 2011 and 2019.

### Features (Independent Variables)

Similar to our previous study [23], we examined 234 candidate features describing a wide variety of characteristics. Table S1 of Multimedia Appendix 1 describes these features calculated on the structured attributes in our data set, with the 71 new features not used in our previous study [23] marked in italics. Throughout this paper, every mention of the number of a particular kind of items such as medications counts multiplicity whenever the word differing is absent. For instance, consider a patient who was ordered medications twice in a given year. The first time, medications 1 and 2 were ordered for the patient. The second time, medications 2 and 3 were ordered for the patient. Then, the total number of medications ordered for the patient in this year was 4. The total number of differing medications ordered for the patient in this year was 3.

Every input data instance to the predictive model addresses a unique index year and patient pair and is used to forecast the patient’s outcome in the subsequent 12 months, that is, the 12 months after the end of the index year. For that pair, we computed the patient’s age and primary care provider (PCP) on the last day of the index year. The PCP identified was the patient’s last PCP recorded in the electronic medical record system on or before the last day of the index year. As Figure 1 shows, adopting the data in the preindex and index years, we computed 1 feature: the percentage of the PCP’s patients with asthma in the preindex year incurring asthma hospital encounters in the index year. Using the data from 2011 to the index year, we computed 25 features: the number of years from the first encounter related to asthma in the data set, the number of years from the first encounter related to chronic obstructive pulmonary disease in the data set, family history of asthma, 15 features related to the problem list, and 7 allergy features. We derived the other 208 features from the data in the index year.

### Data Analysis

#### Data Preparation

Our UWM data set included peak expiratory flow values, which were absent in the Intermountain Healthcare data set adopted in our previous study [23]. Adopting the lower and upper bounds supplied by a clinical expert in our team, we deemed all peak expiratory flow values more than 700 biologically implausible. Adopting the data preparation approach used in our previous paper [23] and this criterion, we pinpointed biologically implausible values, marked them missing, and normalized data. As the outcome of interest came from the subsequent year, our data set included 8 years of effective data (2011-2018) over the 9-year period of 2011-2019. To be consistent with future model use in practice, we used the 2011-2017 data to train the models and the 2018 data to evaluate model performance.

#### Performance Metrics

As presented in Table 1 and the formulas below, we evaluated model performance using 6 standard metrics: accuracy, AUC, sensitivity, specificity, positive predictive value (PPV), and negative predictive value (NPV).

Accuracy = (TP + TN)/(TP + TN + FP + FN) **(1)**

Sensitivity = TP/(TP + FN) **(2)**

Specificity = TN/(TN + FP) **(3)**

PPV = TP/(TP + FP) **(4)**

NPV = TN/(TN + FN) **(5)**

Here, TP stands for true positive. TN stands for true negative. FP stands for false positive. FN stands for false negative.

**Table 1:** The confusion matrix.

Outcome class	Future asthma hospital encounters	No future asthma hospital encounter
Forecasted future asthma hospital encounters	True positive	False positive
Forecasted no future asthma hospital encounter	False negative	True negative

We performed a 1000-fold bootstrap analysis [31] to calculate 95% CIs for the 6 performance measures. For instance, we computed our final UWM model’s performance measures for each bootstrap sample of the 2018 data. The 2.5th and 97.5th percentiles of the 1000 values we obtained for every performance metric gave the 95% CI of the corresponding performance measure. We rendered the receiver operating characteristic curve to show the sensitivity-specificity tradeoff.

#### Classification Algorithms

As in our previous paper [23], our predictive models were built using Waikato Environment for Knowledge Analysis (Weka) Version 3.9 [32]. Weka is a core open-source software package for data mining and machine learning. It integrates a large number of popular feature selection techniques and machine learning algorithms. We checked the extreme gradient boosting (XGBoost) machine learning classification algorithm [33] implemented in the software package XGBoost4J [34] and the 39 native classification algorithms in Weka listed in our previous paper’s web-based multimedia appendix [23]. As an efficient and scalable realization of gradient boosting, XGBoost is a form of an ensemble of decision trees. As XGBoost accepts only numerical features, we used one-hot encoding to transform categorical features into numerical features before giving them to XGBoost. We used the 2011-2017 training data and the automatic machine learning model selection method developed in our previous work [35] to automatically select the feature selection technique, classification algorithm, data balancing method for handling imbalanced data, and hyperparameter values among all of the pertinent ones. On average, our method can reduce the model error rate by 11% and search time by 28 times compared with the modern Auto-WEKA automatic machine learning model selection method [35,36].

This study mainly evaluated our modeling strategy’s generalizability to the UWM by using the UWM training set to train multiple models and then checking their performance on the UWM test set. In addition, we conducted 2 experiments to evaluate the generalizability of our models across health systems.

#### Evaluating the Generalizability of Our Intermountain Healthcare Model to the UWM

In the first experiment, we evaluated the generalizability of our Intermountain Healthcare model to the UWM. Previously, we developed both a simplified model and a full model on the Intermountain Healthcare data set [23]. Our simplified Intermountain Healthcare model uses the top 21 features whose importance values calculated by XGBoost on that data set are ≥0.01 [23]. Compared with our full Intermountain Healthcare model using 142 features, our simplified Intermountain Healthcare model retained nearly all of its predictive power. Our UWM data set contained the top 21 features and missed some other features adopted in our full Intermountain Healthcare model. We evaluated the performance of our simplified Intermountain Healthcare model on the UWM test set twice. The first time, we retrained our simplified Intermountain Healthcare model on the UWM training set. The second time, we did not perform retraining and directly applied our original simplified Intermountain Healthcare model trained on the Intermountain Healthcare training set.

#### Evaluating the Generalizability of Our UWM Model to Intermountain Healthcare

In the second experiment, we evaluated the generalizability of our UWM model to Intermountain Healthcare. We used a simplified UWM model, which used only the top features whose importance values calculated by XGBoost on the UWM training set were ≥0.01. For any top feature that was newly introduced in this study and was not used in our previous study [23], we computed the feature on the Intermountain Healthcare data set. We evaluated our simplified UWM model’s performance on the Intermountain Healthcare test set twice. The first time, we retrained our simplified UWM model using the Intermountain Healthcare training set. The second time, we did not perform retraining and directly applied our simplified UWM model trained on the UWM training set.

## Results

### Demographic and Clinical Characteristics of Our Patient Cohort

Each data instance addresses a unique index year and patient pair. Tables 2 and 3 show the demographic and clinical characteristics of our UWM patient cohort during 2011-2017 and 2018, respectively. The characteristics were similar across the 2 periods. During 2011-2017 and 2018, 1.74% (1184/68,244) and 1.49% (218/14,644) of data instances were linked to asthma hospital encounters in the subsequent 12 months, respectively.

**Table 2:** Demographic and clinical characteristics of patients with asthma at the University of Washington Medicine during 2011-2017.

Characteristic			Data instances (N=68,244), n (%)		Data instances connecting to asthma hospital encounters in the subsequent 12 months (n=1184), n (%)		Data instances connecting to no asthma hospital encounter in the subsequent 12 months (n=67,060), n (%)
**Age (years)**							
	<40	23,459 (34.38)		466 (39.36)		22,993 (34.29)	
	40-65	33,889 (49.66)		583 (49.24)		33,306 (49.67)	
	>65	10,896 (15.97)		135 (11.40)		10,761 (16.05)	
**Gender**							
	Male	24,198 (35.46)		551 (46.54)		23,647 (35.26)	
	Female	44,046 (64.54)		633 (53.46)		43,413 (64.74)	
**Race**							
	American Indian or Alaska native	1358 (1.99)		32 (2.70)		1326 (1.98)	
	Asian	5721 (8.38)		96 (8.11)		5625 (8.39)	
	Black or African American	8420 (12.34)		520 (43.92)		7900 (11.78)	
	Native Hawaiian or other Pacific islander	673 (0.99)		14 (1.18)		659 (0.98)	
	White	47,747 (69.97)		507 (42.82)		47,240 (70.44)	
	Unknown or not reported	4325 (6.34)		15 (1.27)		4310 (6.43)	
**Ethnicity**							
	Hispanic	3526 (5.17)		82 (6.93)		3444 (5.14)	
	Non-Hispanic	56,309 (82.51)		1062 (89.70)		55,247 (82.38)	
	Unknown or not reported	8409 (12.32)		40 (3.38)		8369 (12.48)	
**Insurance**							
	Private	40,009 (58.63)		424 (35.81)		39,585 (59.03)	
	Public	28,787 (42.18)		756 (63.85)		28,031 (41.80)	
	Self-paid or charity	1366 (2.00)		65 (5.49)		1301 (1.94)	
**Number of years from the first encounter related to asthma in the data set**							
	≤3	60,873 (89.20)		986 (83.28)		59,887 (89.30)	
	>3	7371 (10.80)		198 (16.72)		7173 (10.70)	
**Asthma medication prescription**							
	Inhaled corticosteroid	28,889 (42.33)		626 (52.88)		28,263 (42.15)	
	Inhaled corticosteroid and long-acting β-2 agonist combination	22,015 (32.26)		499 (42.15)		21,516 (32.08)	
	Leukotriene modifier	8171 (11.97)		201 (16.98)		7970 (11.88)	
	Long-acting β-2 agonist	12,293 (18.01)		374 (31.59)		11,919 (17.77)	
	Mast cell stabilizer	47 (0.07)		4 (0.34)		43 (0.06)	
	Short-acting inhaled β-2 agonist	47,808 (70.05)		1010 (85.30)		46,798 (69.79)	
	Systemic corticosteroid	18,699 (27.40)		614 (51.86)		18,085 (26.97)	
**Comorbidity**							
	Allergic rhinitis	11,449 (16.78)		172 (14.53)		11,277 (16.82)	
	Anxiety or depression	19,885 (29.14)		372 (31.42)		19,513 (29.10)	
	Bronchopulmonary dysplasia	1 (0)		0 (0)		1 (0)	
	Chronic obstructive pulmonary disease	3826 (5.61)		133 (11.23)		3693 (5.51)	
	Cystic fibrosis	61 (0.09)		1 (0.08)		60 (0.09)	
	Eczema	3891 (5.70)		66 (5.57)		3825 (5.70)	
	Gastroesophageal reflux	12,291 (18.01)		238 (20.10)		12,053 (17.97)	
	Obesity	7845 (11.50)		177 (14.95)		7668 (11.43)	
	Sinusitis	7261 (10.64)		89 (7.52)		7172 (10.69)	
	Sleep apnea	4556 (6.68)		88 (7.43)		4468 (6.66)	
**Smoking status**							
	Current smoker	14,081 (20.63)		255 (21.54)		13,826 (20.62)	
	Former smoker	15,530 (22.76)		221 (18.67)		15,309 (22.83)	
	Never smoker or unknown	38,633 (56.61)		708 (59.80)		37,925 (56.55)	

**Table 3:** Demographic and clinical characteristics of patients with asthma at the University of Washington Medicine in 2018.

Characteristic			Data instances (N=14,644), n (%)		Data instances connecting to asthma hospital encounters in the subsequent 12 months (n=218), n (%)		Data instances connecting to no asthma hospital encounter in the subsequent 12 months (n=14,426), n (%)
**Age (years)**							
	<40	4823 (32.9)		77 (35.3)		4746 (32.9)	
	40-65	6794 (46.4)		111 (50.9)		6683 (46.3)	
	>65	3027 (20.7)		30 (13.8)		2997 (20.8)	
**Gender**							
	Male	5238 (35.8)		100 (45.9)		5138 (35.6)	
	Female	9406 (64.2)		118 (54.2)		9288 (64.4)	
**Race**							
	American Indian or Alaska native	281 (1.9)		8 (3.7)		273 (1.9)	
	Asian	1325 (9.1)		18 (8.7)		1307 (9.1)	
	Black or African American	1570 (10.7)		79 (36.2)		1491 (10.3)	
	Native Hawaiian or other Pacific islander	131 (0.9)		2 (0.9)		129 (0.9)	
	White	10,213 (69.7)		110 (50.5)		10,103 (70)	
	Unknown or not reported	1124 (7.7)		1 (0.5)		1123 (7.8)	
**Ethnicity**							
	Hispanic	850 (5.8)		20 (9.2)		830 (5.7)	
	Non-Hispanic	12,566 (85.8)		196 (89.9)		12,370 (85.7)	
	Unknown or not reported	1228 (8.4)		2 (0.9)		1226 (8.5)	
**Insurance**							
	Private	10,800 (73.7)		108 (49.5)		10,692 (74.1)	
	Public	8023 (54.8)		182 (83.5)		7841 (54.3)	
	Self-paid or charity	484 (3.3)		25 (11.5)		459 (3.2)	
**Number of years from the first encounter related to asthma in the data set**							
	≤3	10,566 (72.1)		124 (56.9)		10,442 (72.4)	
	>3	4078 (27.8)		94 (43.1)		3984 (27.6)	
**Asthma medication prescription**							
	Inhaled corticosteroid	6177 (42.2)		108 (49.5)		6069 (42.1)	
	Inhaled corticosteroid and long-acting β-2 agonist combination	4508 (30.8)		83 (38.1)		4425 (30.7)	
	Leukotriene modifier	2176 (14.9)		46 (21.1)		2130 (14.77)	
	Long-acting β-2 agonist	2518 (17.2)		62 (28.4)		2456 (17.02)	
	Mast cell stabilizer	14 (0.1)		1 (0.5)		13 (0.09)	
	Short-acting inhaled β-2 agonist	9704 (66.3)		164 (75.2)		9540 (66.1)	
	Systemic corticosteroid	4163 (28.4)		120 (55.1)		4043 (28)	
**Comorbidity**							
	Allergic rhinitis	2095 (14.3)		26 (11.9)		2069 (14.3)	
	Anxiety or depression	4346 (29.7)		62 (28.4)		4284 (29.7)	
	Bronchopulmonary dysplasia	4 (0)		0 (0)		4 (0)	
	Chronic obstructive pulmonary disease	932 (6.4)		30 (13.8)		902 (6.2)	
	Cystic fibrosis	17 (0.1)		0 (0)		17 (0.1)	
	Eczema	743 (5.1)		11 (5.1)		732 (5.1)	
	Gastroesophageal reflux	2657 (18.1)		46 (21.1)		2611 (18.1)	
	Obesity	1604 (10.9)		25 (11.5)		1579 (10.9)	
	Sinusitis	1372 (9.4)		15 (6.9)		1357 (9.4)	
	Sleep apnea	1499 (10.2)		24 (11.0)		1475 (10.2)	
**Smoking status**							
	Current smoker	3242 (22.1)		49 (22.5)		3193 (22.1)	
	Former smoker	3494 (23.9)		41 (18.8)		3453 (23.9)	
	Never smoker or unknown	7908 (54.0)		128 (58.7)		7780 (53.9)	

As the Chi-square 2-sample test showed, for both the 2011-2017 and 2018 data, the data instances connecting to future asthma hospital encounters and those connecting to no future asthma hospital encounter exhibited the same distribution for anxiety or depression occurrence (*P*=.74 for the 2018 data and *P*=.09 for the 2011-2017 data), bronchopulmonary dysplasia occurrence (*P*=.99), cystic fibrosis occurrence (*P*=.99), eczema occurrence (*P*=.99 for the 2018 data and *P*=.90 for the 2011-2017 data), gastroesophageal reflux occurrence (*P*=.29 for the 2018 data and *P*=.06 for the 2011-2017 data), and sleep apnea occurrence (*P*=.79 for the 2018 data and *P*=.32 for the 2011-2017 data). These 2 sets of data instances exhibited differing distributions for gender (*P*=.002 for the 2018 data and *P*<.001 for the 2011-2017 data), ethnicity (*P*<.001), insurance category (*P*<.001), race (*P*<.001), systemic corticosteroid prescription (*P*<.001), inhaled corticosteroid prescription (*P*=.02 for the 2018 data and *P*<.001 for the 2011-2017 data), inhaled corticosteroid and long-acting β-2 agonist combination prescription (*P*=.02 for the 2018 data and *P*<.001 for the 2011-2017 data), short-acting inhaled β-2 agonist prescription (*P*=.006 for the 2018 data and *P*<.001 for the 2011-2017 data), long-acting β-2 agonist prescription (*P*<.001), leukotriene modifier prescription (*P*=.01 for the 2018 data and *P*<.001 for the 2011-2017 data), and chronic obstructive pulmonary disease occurrence (*P*<.001). For the 2011-2017 data, these 2 sets of data instances exhibited differing distributions for mast cell stabilizer prescription (*P*=.003), obesity occurrence (*P*<.001), sinusitis occurrence (*P*<.001), allergic rhinitis occurrence (*P*=.04), and smoking status (*P*=.003). For the 2018 data, these 2 sets of data instances exhibited the same distribution for mast cell stabilizer prescription (*P*=.52), obesity occurrence (*P*=.89), sinusitis occurrence (*P*=.25), allergic rhinitis occurrence (*P*=.36), and smoking status (*P*=.19).

As the Cochran-Armitage trend test [37] showed, the data instances connecting to future asthma hospital encounters and those connecting to no future asthma hospital encounter exhibited the same distribution for age (*P*=.06) in the 2018 data and differing distributions for age (*P*<.001) in the 2011-2017 data. With regard to the 2018 and 2011-2017 data, these 2 sets of data instances exhibited differing distributions for the number of years from the first encounter related to asthma in the data set (*P*<.001).

Table 4 shows the number of patients with asthma and their number of visits in each year between 2011 and 2018.

**Table 4:** The number of patients with asthma and their number of visits in each year between 2011 and 2018.

Year	Number of patients with asthma	Number of visits by patients with asthma
2011	6852	32,910
2012	7768	40,730
2013	7754	39,385
2014	9785	58,953
2015	10,587	69,285
2016	12,072	78,605
2017	13,426	87,403
2018	14,644	94,875

### Classification Algorithm and Features Adopted by Our Final UWM Model

Our automatic machine learning model selection method [35] selected the XGBoost classification algorithm [33]. XGBoost is a form of an ensemble of decision trees that can naturally deal with missing feature values. As described in Hastie et al [38] in detail, XGBoost automatically calculates the importance value of each feature based on its apportioned contribution to the model. Our final UWM model was formed using XGBoost and 71 features displayed in descending order of their importance values in Table S2 of Multimedia Appendix 1. XGBoost automatically removed the other features because they had no additional predictive power.

### Performance Measures Yielded by Our Final UWM Model

**Figure 2:**
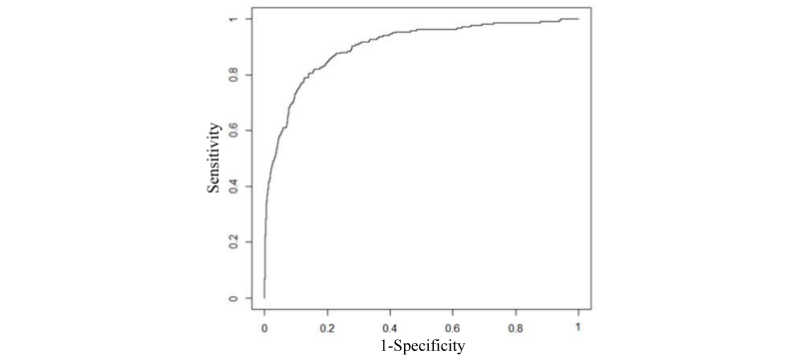
The receiver operating characteristic curve of our final University of Washington Medicine model.

On the UWM test set, our final model yielded an AUC of 0.902 (95% CI 0.879-0.924). Figure 2 shows the receiver operating characteristic curve of the model. Table 5 lists the model’s performance measures when the cutoff point for making binary classification was placed at different top percentages of patients with asthma with the largest forecasted risk. When the cutoff point was placed at the top 10% (1464/14,644), the model yielded an accuracy of 90.6% (13,268/14,644; 95% CI 90.13-91.06), a sensitivity of 70.2% (153/218; 95% CI 63.8-76.0), a specificity of 90.91% (13,115/14,426; 95% CI 90.45-91.38), a PPV of 10.45% (153/1464; 95% CI 8.90-11.97), and an NPV of 99.51% (13,115/13,180; 95% CI 99.39-99.62). Table 6 presents the confusion matrix of the model in this case.

Several features, such as a family history of asthma, were calculated on 2 or more years of data. When we dropped these features and checked solely those features calculated on 1 year of data, the AUC of the model decreased from 0.902 to 0.899. If we used only the top 17 features in Table S2 of Multimedia Appendix 1 whose importance values are ≥0.01 and ignored the other 217 features, the model’s AUC decreased from 0.902 to 0.898 (95% CI 0.874-0.919). In this case, when we placed the cutoff point for making binary classification at the top 10% (1464/14,644) of patients with asthma with the largest forecasted risk, the model’s accuracy decreased from 90.6% (13,268/14,644) to 90.59% (13,266/14,644; 95% CI 90.11-91.06), sensitivity decreased from 70.2% (153/218) to 69.7% (152/218; 95% CI 63.6-75.5), specificity remained at 90.91% (13,114/14,426; 95% CI 90.42-91.37), PPV decreased from 10.45% (153/1464) to 10.38% (152/1464; 95% CI 8.82-11.97), and NPV decreased from 99.51% (13,115/13,180) to 99.5% (13,114/13,180; 95% CI 99.38-99.61).

**Table 5:** Our final UWM model’s performance measures when the cutoff point for making binary classification was placed at different top percentages of patients with asthma with the largest forecasted risk.

Top percentage of patients with asthma with the largest forecasted risk (%)	Accuracy (N=14,644), n (%)	Sensitivity (N=218), n (%)	Specificity (N=14,426), n (%)	PPV^a^		NPV^b^	
				n (%)	N	n (%)	N
1	14,410 (98.4)	65 (29.8)	14,345 (99.4)	65 (44.5)	146	14,345 (98.9)	14,498
2	14,316 (97.8)	91 (41.7)	14,225 (98.6)	91 (31.2)	292	14,225 (99.1)	14,352
3	14,193 (96.9)	103 (47.3)	14,090 (97.7)	103 (23.5)	439	14,090 (99.2)	14,205
4	14,061 (96)	110 (50.5)	13,951 (96.7)	110 (18.8)	585	13,951 (99.2)	14,059
5	13,936 (95.2)	121 (55.5)	13,815 (95.8)	121 (16.5)	732	13,815 (99.3)	13,912
6	13,806 (94.3)	129 (59.2)	13,677 (94.8)	129 (14.7)	878	13,677 (99.3)	13,766
7	13,667 (93.3)	133 (61)	13,534 (93.8)	133 (13)	1025	13,534 (99.4)	13,619
8	13,529 (92.4)	137 (62.8)	13,392 (92.8)	137 (11.7)	1171	13,392 (99.4)	13,473
9	13,411 (91.6)	151 (69.3)	13,260 (91.9)	151 (11.5)	1317	13,260 (99.5)	13,327
10	13,268 (90.6)	153 (70.2)	13,115 (90.9)	153 (10.5)	1464	13,115 (99.5)	13,180
15	12,576 (85.9)	173 (79.4)	12,403 (86)	173 (7.9)	2196	12,403 (99.6)	12,448
20	11,860 (81)	181 (83)	11,679 (81)	181 (6.2)	2928	11,679 (99.7)	11,716
25	11,147 (76.1)	191 (87.6)	10,956 (75.9)	191 (5.2)	3661	10,956 (99.7)	10,983

^a^PPV: positive predictive value.

^b^NPV: negative predictive value.

**Table 6:** The confusion matrix of our final University of Washington Medicine model when the cutoff point for making binary classification was placed at the top 10% (1464/14,644) of patients with asthma with the largest forecasted risk.

Outcome class	Future asthma hospital encounter, n	No future asthma hospital encounter, n
Forecasted future asthma hospital encounters	153	1311
Forecasted no future asthma hospital encounter	65	13,115

### Performance Measures Yielded by Our Simplified Intermountain Healthcare Model on UWM Data

For our original simplified Intermountain Healthcare model trained on the Intermountain Healthcare training set [23], when we did not retrain the model and applied the model directly to the UWM test set, the model yielded an AUC of 0.861 (95% CI 0.835-0.885). When we placed the cutoff point for making binary classification at the top 10% (1464/14,644) of patients with asthma with the largest forecasted risk, the model yielded an accuracy of 90.29% (13,222/14,644; 95% CI 89.81-90.77), a sensitivity of 59.6% (130/218; 95% CI 53.4-65.7), a specificity of 90.75% (13,092/14,426; 95% CI 90.28-91.20), a PPV of 8.88% (130/1464; 95% CI 7.46-10.34), and an NPV of 99.33% (13,092/13,180; 95% CI 99.20-99.46).

After we used the UWM training set to retrain our simplified Intermountain Healthcare model [23], the retrained model yielded on the UWM test set an AUC of 0.874 (95% CI 0.848-0.896). When we placed the cutoff point for making binary classification at the top 10% (1464/14,644) of patients with asthma with the largest forecasted risk, the model yielded an accuracy of 90.34% (13,230/14,644; 95% CI 89.85-90.80), a sensitivity of 61.5% (134/218; 95% CI 54.6-67.7), a specificity of 90.78% (13,096/14,426; 95% CI 90.32-91.23), a PPV of 9.15% (134/1464; 95% CI 7.62-10.66), and an NPV of 99.36% (13,096/13,180; 95% CI 99.22-99.49).

### Performance Measures Yielded by Our Simplified UWM Model on Intermountain Healthcare Data

Our simplified UWM model used only the top 17 features with importance values of ≥0.01. For our simplified UWM model trained on the UWM training set, when we did not retrain the model and applied the model directly to the Intermountain Healthcare test set, the model yielded an AUC of 0.814 (95% CI 0.798-0.830). When we placed the cutoff point for making binary classification at the top 10% (1926/19,256) of patients with asthma with the largest forecasted risk, the model yielded an accuracy of 89.76% (17,285/19,256; 95% CI 89.32-90.18), a sensitivity of 47.2% (383/812; 95% CI 43.8-50.6), a specificity of 91.64% (16,902/18,444; 95% CI 91.24-92.03), a PPV of 19.90% (383/1925; 95% CI 18.16-21.60), and an NPV of 97.52% (16,902/17,331; 95% CI 97.28-97.75).

After we used the Intermountain Healthcare training set to retrain our simplified UWM model, the retrained model yielded on the Intermountain Healthcare test set an AUC of 0.846 (95% CI 0.831-0.859). When we placed the cutoff point for making binary classification at the top 10% (1926/19,256) of patients with asthma with the largest forecasted risk, the model yielded an accuracy of 90.11% (17,351/19,256; 95% CI 89.64-90.56), a sensitivity of 51.2% (416/812; 95% CI 47.6-54.5), a specificity of 91.82% (16,935/18,444; 95% CI 91.43-92.21), a PPV of 21.62% (416/1,925; 95% CI 19.81-23.41), and an NPV of 97.72% (16,935/17,331; 95% CI 97.48-97.93).

## Discussion

### Principal Findings

We built a model on UWM data to forecast asthma hospital encounters of patients with asthma in the subsequent 12 months. Table 7 reveals that our final UWM model yielded an AUC that was higher than the previously reported AUC of every existing model [2,9-23], that is, our modeling strategy of examining many candidate features to enhance model accuracy showed excellent generalizability to the UWM. After further optimization to boost its accuracy and automatically provide explanations of its predictions [39,40] to allow clinical interpretability, our UWM model could be used to facilitate efficient and effective allocation of asthma care management resources to improve outcomes.

In Table S2 of Multimedia Appendix 1, both the 5 most important features and multiple other features within the top 17 indicate a loss of asthma control. It is important to note that the loss of asthma control could be partly because of factors not well captured in our data, such as socioeconomic circumstances, variable management practices among providers, access to subspecialty clinicians, and nonadherence to medications and treatments. Variable asthma severity across patients over time also influences this process.

We checked 234 candidate features. Our final UWM model used 30.3% (71/234) of them. Despite being correlated with the outcome, many unused features had no extra predictive power on the UWM data set over the features adopted in our final UWM model.

For our original simplified Intermountain Healthcare model trained on the Intermountain Healthcare training set [23], when we did not retrain the model on the UWM data and directly applied the model, the model yielded an AUC of 0.861 on the UWM test set. This AUC is 0.041 lower than our final UWM model’s AUC, but is still larger than the previously reported AUC of every existing model for forecasting future hospitalizations and ED visits of patients with asthma (Table 7). Therefore, our simplified Intermountain Healthcare model showed excellent generalizability to the UWM.

Compared with our full UWM model using 71 features, our simplified UWM model retained nearly all of its predictive power. For our simplified UWM model trained on the UWM training set, when we did not retrain the model on the Intermountain Healthcare data and directly applied the model, the model yielded an AUC of 0.814 on the Intermountain Healthcare test set. This AUC is 0.045 lower than our full Intermountain Healthcare model’s AUC but is still larger than the previously reported AUC of every existing model developed by others for forecasting future hospitalizations and ED visits of patients with asthma (Table 7). Therefore, our simplified UWM model shows excellent generalizability to Intermountain Healthcare.

**Table 7:** A comparison of our final University of Washington Medicine model and several existing models for forecasting future hospitalizations and emergency department (ED) visits of patients with asthma.

Model	Prediction target	Number of data instances	Number of features the model adopted	Classification algorithm	Sensitivity (%)	Specificity (%)	PPV^a^ (%)	NPV^b^ (%)	AUC^c^
Our final UWM model	Asthma hospital encounters	82,888	71	XGBoost^d^	70.2	90.91	10.45	99.51	0.902
Our Intermountain Healthcare model [23]	Asthma hospital encounters	334,564	142	XGBoost	53.69	91.93	22.65	97.83	0.859
Loymans et al [9]	Asthma exacerbation	611	7	Logistic regression	—^e^	—	—	—	0.8
Schatz et al [10]	Asthma-induced hospitalization in children	4197	5	Logistic regression	43.9	89.8	5.6	99.1	0.781
Schatz et al [10]	Asthma-induced hospitalization in adults	6904	3	Logistic regression	44.9	87	3.9	99.3	0.712
Eisner et al [11]	Asthma-induced hospitalization	2858	1	Logistic regression	—	—	—	—	0.689
Eisner et al [11]	Asthma-induced ED visit	2415	3	Logistic regression	—	—	—	—	0.751
Sato et al [12]	Severe asthma exacerbation	78	3	Classification and regression tree	—	—	—	—	0.625
Miller et al [14]	Asthma hospital encounters	2821	17	Logistic regression	—	—	—	—	0.81
Yurk et al [16]	Lost day or hospital encounters for asthma	4888	11	Logistic regression	77	63	82	56	0.78
Lieu et al [2]	Asthma-induced hospitalization	16,520	7	Proportional-hazards regression	—	—	—	—	0.79
Lieu et al [2]	Asthma-induced ED visit	16,520	7	Proportional-hazards regression	—	—	—	—	0.69
Lieu et al [18]	Asthma hospital encounters	7141	4	Classification and regression tree	49	83.6	18.5	—	—
Schatz et al [19]	Asthma hospital encounters	14,893	4	Logistic regression	25.4	92	22	93.2	0.614
Forno et al [21]	Severe asthma exacerbation	615	17	Scoring	—	—	—	—	0.75
Xiang et al [22]	Asthma exacerbation	31,433	—	Recurrent neural network	—	—	—	—	0.70

^a^PPV: positive predictive value.

^b^NPV: negative predictive value.

^c^AUC: area under the receiver operating characteristic curve.

^d^XGBoost: extreme gradient boosting.

^e^The initial paper showing the model did not give the performance measure.

### Comparison With the Previous Work

Researchers have built multiple models to forecast future hospitalizations and ED visits of patients with asthma [2,9-23]. Table 7 compares our final UWM model with these models, which cover all the relevant models described in the systematic review by Loymans et al [17]. The final UWM model’s AUC was 0.902. The AUC of our Intermountain Healthcare model was 0.859. All other existing models have a previously reported AUC 0.81 [2,9-22], which is lower than our final UWM model’s AUC by at least 0.091.

It is important to consider the prevalence of the outcome of interest when comparing the performance of different predictive models. Compared with other existing models, the model by Yurk et al [16] achieved a higher sensitivity and PPV mainly because it adopted a different prediction target: asthma hospital encounters or at least 1 day lost for diminished activities or missing work for asthma. This prediction target had a 54% prevalence rate in patients with asthma and was therefore easier to forecast. If the model by Yurk et al [16] were used to forecast asthma hospital encounters, an outcome that had a <2% prevalence rate in patients with asthma, the model’s sensitivity and PPV would likely drop.

The recurrent neural network model by Xiang et al [22] reached a low AUC of 0.7, mainly because it used mostly inpatient data with little outpatient data; adopted only 3 types of attributes: medication, diagnosis, and demographics; and did not merge individual asthma medications into asthma medication categories such as nebulizers and short-acting β-2 agonists, that is, the low AUC does not prove that the recurrent neural network is ineffective at predicting asthma outcomes, but is mainly because of incomplete data and insufficient feature modeling. In comparison, to build our final UWM model, we used both inpatient and outpatient data, adopted many types of attributes, and merged individual asthma medications into asthma medication categories to better capture and model the relationship among different asthma medications.

Excluding the model by Yurk et al [16], every existing published model has a sensitivity 53.69%, which is significantly lower than our final UWM model’s sensitivity of 70.2%. For patients with asthma who will have future asthma hospital encounters, sensitivity is the percentage of them identified by the model. The difference in sensitivity can have a significant impact on health care use. Owing to the high prevalence rate of asthma, for every 10% increase in the identified percentage of patients with asthma who would have future asthma hospital encounters, up to 7759 more hospitalizations and 71,074 more ED visits could be avoided in the United States each year with effective care management [1,4-7].

The prevalence rate of targeted poor outcomes greatly impacts the PPV of any predictive model [41]. In our UWM test data set, 1.49% (218/14,644) of patients with asthma had future asthma hospital encounters. When we placed the cutoff point for making binary classification at the top 10% (1464/14,644) of patients with asthma with the largest forecasted risk, an impeccable model in theory would yield the highest possible PPV of 14.89% (218/1464). Our final UWM model yielded a PPV of 10.45% (153/1464), which is 70.18% of the highest possible PPV in theory. In comparison, our Intermountain Healthcare model achieved a PPV of 22.65% [23]. This is 53.69% of the highest possible PPV that an impeccable model in theory would yield on the Intermountain Healthcare test set. The model by Lieu et al [18] yielded a PPV of 18.5% on a data set where 6.9% of patients with asthma had future asthma hospital encounters. The model by Schatz et al [19] yielded a PPV of 22% on a data set where 6.5% of patients with asthma had future asthma hospital encounters. Compared with our case with UWM, both populations have a higher prevalence of asthma hospital encounters, which allows the PPV to be higher. Excluding these PPVs and the PPV by the Yurk et al [16] model, no other existing published model’s PPV exceeds 5.6%.

Our final UWM model and our Intermountain Healthcare model [23] have similar top features with importance values of ≥0.01. In both models, many top features are related to previous ED visits and asthma medications. We did not identify several candidate features at the time of constructing our Intermountain Healthcare model. They appeared as top features and affected the ranks and importance values of the other top features in our final UWM model.

Differing models in Table 7 were built using different patient cohorts and used similar but not necessarily identical prediction targets. Some features used in the models built by other researchers, such as certain features computed from patient-reported outcomes and patient surveys, are unavailable in our UWM data set. Therefore, we were unable to show the performance measures that the models built by other researchers would achieve on our UWM data set. However, we are confident that the techniques used in this study improved prediction accuracy. Our final UWM model was built using a state-of-the-art machine learning algorithm, XGBoost. Compared with statistical methods such as logistic regression, machine learning can enhance prediction accuracy with less strict assumptions on data distribution [8,42,43]. Compared with the models built by other researchers, our final UWM model was built using more patients and a more extensive set of candidate features constructed with careful feature engineering, both of which are known to often help improve prediction accuracy [24-27,32]. As partial evidence for this, we built predictive models for asthma hospital encounters using data from 3 health care systems: UWM, Intermountain Healthcare [23], and Kaiser Permanente Southern California [44]. For each of the 3 health care systems, we started model building with approximately 20 candidate features and obtained unsatisfactory accuracy. This motivated us to examine several hundred candidate features. Ultimately, for each of the 3 health care systems, we built a model with an AUC that is higher than all of the AUCs other researchers previously reported in the literature for forecasting asthma hospital encounters [23,44]. This demonstrates the generalizability of our modeling strategy for forecasting asthma hospital encounters.

### Considerations Concerning the Potential Clinical Use

Our final UWM model has an AUC that is higher than all of the AUCs previously reported in the literature for forecasting asthma hospital encounters, but still had a seemingly low PPV of 10.4% (153/1464). Nevertheless, this model could be valuable in clinical care. First, health care systems such as UWM, Intermountain Healthcare, and Kaiser Permanente Northern California [2] use proprietary models to allocate asthma care management resources. These models and the models that were formerly built by others have similar performance measures. Our final UWM model has an AUC that is higher than the previously reported AUCs of all these models.

Second, as explained earlier, even an impeccable model in theory would reach a low PPV because the poor outcome of interest has a low prevalence rate in our data set. For such an outcome, sensitivity better reflects the model’s potential clinical value than PPV. Our final UWM model had a higher sensitivity than the previously reported sensitivity of every existing model using a comparable prediction target. It is important to note that while asthma hospital encounters have an overall low prevalence rate in the population of patients with asthma, they have significant financial and clinical impacts at both the population and individual patient levels.

Third, a PPV of 10.45% (153/1464) is useful for identifying high-risk patients with asthma to receive low-cost preventive interventions. The following are 4 examples of such interventions: training the patient to record a diary about environmental triggers, coaching the patient to use an asthma inhaler correctly, coaching the patient to use a peak flow meter correctly and giving it to the patient to self-monitor symptoms at home, and asking a nurse to do extra follow-up phone calls with the patient or the patient’s caregiver. These interventions could have a significant impact on patient outcomes.

The final UWM model used 71 features. Reducing the number of features could ease the clinical deployment of our model. To this end, if a minor decrease in prediction accuracy could be tolerated, one could adopt the top few features whose importance values are greater than a given threshold, such as 0.01, and drop the other features. The importance value of a feature varies across health care systems. Ideally, the importance values of the features should first be calculated on a data set from the target health care system before choosing the features to retain.

As is typical with complex machine learning models, an XGBoost model using many features is difficult to interpret. This can limit clinical understandability and adoption, particularly by clinicians who are resistant to using automated tools. In the future, we plan to adopt our previously developed method [39,40] to automatically explain the prediction results of our final UWM model.

The final UWM model was constructed using XGBoost [33]. For binary classification of imbalanced data, XGBoost leverages a hyperparameter, scale_pos_weight, to balance the weights of the 2 outcome classes [45]. To maximize the AUC of our UWM model, our automatic model selection method [35] altered scale_pos_weight to a nondefault value to balance the 2 outcome classes [46]. This incurs a side effect of significantly shrinking the model’s forecasted probabilities of having future asthma hospital encounters to values much less than the actual probabilities [46]. This does not preclude us from choosing the top few percent of patients with asthma with the greatest forecasted risk to receive various preventive interventions. To prevent this side effect from occurring, we could retain scale_pos_weight at its default value of 1 without doing any balancing. As a tradeoff, the AUC of the model would decrease from 0.902 to 0.885 (95% CI 0.861-0.907); however, this decreased AUC is still larger than all of the AUCs previously reported in the literature for forecasting asthma hospital encounters.

### Limitations

This study has at least 4 limitations that could be interesting topics for future work, as follows:

1. It is possible to further increase the model accuracy by using features other than those checked in this study. For example, features derived from environmental and physiological data gathered by intelligent wearable devices can have this potential.
2. This study used purely structured data and checked only nondeep learning classification algorithms. It is possible to further increase the model accuracy by using deep learning as well as features derived from unstructured clinical notes using natural language processing techniques [40,47].
3. Our UWM data set contained no data on patients’ health care use outside of UWM. Therefore, we limited the prediction target to asthma hospital encounters at UWM instead of asthma hospital encounters anywhere. In addition, the features we checked were derived from patients’ incomplete administrative and clinical data [48-51]. It would be worth investigating how model accuracy would vary if we have more complete administrative and clinical data of patients [52].
4. This study evaluated the generalizability of our modeling strategy to an academic health care system on a single outcome of a complex chronic disease. We recently showed that our modeling strategy also generalizes well to Kaiser Permanente Southern California for the same predictive modeling problem [44]. We plan to investigate our modeling strategy’s generalizability to other diseases, outcomes, and health care systems in the future.

### Conclusions

In the first evaluation of its generalizability to an academic health care system, our modeling strategy of examining many candidate features to enhance prediction accuracy showed excellent generalizability to the UWM and led to a model with an AUC that is higher than all of the AUCs previously reported in the literature for forecasting asthma hospital encounters. After further optimization, our UWM model could be used to facilitate the efficient and effective allocation of asthma care management resources to improve outcomes.

## Appendix

Multimedia Appendix 1 The list of candidate features considered in this study.

## Abbreviations

AUC: area under the receiver operating characteristic curve
ED: emergency department
FN: false negative
FP: false positive
ICD-10: International Classification of Diseases, Tenth Revision
ICD-9: International Classification of Diseases, Ninth Revision
NPV: negative predictive value
PCP: primary care provider
PPV: positive predictive value
TN: true negative
TP: true positive
UWM: University of Washington Medicine
Weka: Waikato Environment for Knowledge Analysis
XGBoost: extreme gradient boosting
