# Supplementary material for: Forecasting Future Asthma Hospital Encounters of Patients With Asthma in an Academic Health Care System: Predictive Model Development and Secondary Analysis Study
Source: J Med Internet Res. 2021 Apr 16;23(4):e22796. doi: 10.2196/22796 (PMC8087967; doi:10.2196/22796)
Supplement: Multimedia Appendix 1 [file jmir_v23i4e22796_app1.pdf]

Appendix

Table 1. The list of candidate features considered in this study.

Feature category	Features
Features on patient demographics	Ethnicity (Hispanic or non-Hispanic); gender; age; marital status (married, single, partnered, divorced, widowed, or separated); race; and language.
Features related to laboratory tests	No. of laboratory tests; no. of laboratory tests having abnormal results; no. of days from the most recent laboratory test; whether an immunoglobulin E (IgE) test was performed; whether the greatest total serum IgE level is abnormally high; the greatest total serum IgE level; the largest percentage of blood eosinophils; and the largest blood eosinophil count.
Features related to vital signs	The mean diastolic blood pressure; the mean heart rate; the mean systolic blood pressure; the highest diastolic blood pressure; the mean temperature; the highest systolic blood pressure; the greatest heart rate; the mean respiratory rate; the greatest respiratory rate; the highest temperature; the mean peripheral capillary oxygen saturation (SpO_2); the lowest SpO_2; *the mean peak expiratory flow; the lowest peak expiratory flow*; the relative change of weight = (the most recently documented weight / the first documented weight - 1) × 100%; the largest body mass index (BMI); and the relative change of BMI = (the most recently documented BMI / the first documented BMI - 1) × 100%.
Features that are related to diagnoses and calculated solely on ICD-9 and ICD-10 diagnosis codes	No. of ICD-9 and ICD-10 diagnosis codes; no. of years from the first encounter related to asthma in the data set; *no. of primary or principal asthma diagnoses; no. of asthma diagnoses; whether the most recent asthma diagnosis is a primary or principal one; the severity of the most recent asthma diagnosis; the severity of the most severe asthma diagnosis; no. of diagnoses of asthma with status asthmaticus; no. of diagnoses of asthma with (acute) exacerbation; the exacerbation severity (uncomplicated, exacerbation, or asthmaticus) of the most recent asthma diagnosis; the greatest exacerbation severity of any asthma diagnosis; no. of days from the most recent asthma diagnosis; no. of days from the most recent diagnosis of asthma with (acute) exacerbation or status asthmaticus; no. of diagnoses of noncompliance with medication regimen; family history of asthma*; chronic obstructive pulmonary disease; no. of years from the first encounter related to chronic obstructive pulmonary disease in the data set; esophagitis; allergic rhinitis; anxiety or depression; ischemic heart disease; eczema; gastroesophageal reflux; sleep apnea; gastrostomy tube; obesity; Alzheimer's or Parkinson's disease; upper respiratory tract infection; decreased tone; increased tone; cystic fibrosis; immunoglobulin A (IgA) deficiency; pneumonia; vocal cord dysfunction; psoriasis; anaphylaxis; vasculitis; cirrhosis; gastrointestinal bleeding; gastrointestinal obstruction; inflammatory bowel disease; mental disorder; breathing abnormality like dyspnea; pregnancy; vitamin D deficiency; myocardial infarction; folate deficiency; congestive heart failure; malignancy; peripheral vascular disease; dementia; peptic ulcer disease; cerebrovascular disease; substance use; rheumatic disease; diabetes with chronic complication; renal disease; diabetes without chronic complication; bronchopulmonary dysplasia; moderate or severe liver disease; mild liver disease; paraplegia or hemiplegia; acquired immunodeficiency syndrome; and metastatic solid tumor.
Features that are related to diagnoses and calculated jointly on ICD-9 and ICD-10 procedure codes along with ICD-9 and ICD-10 diagnosis codes	Tracheostomy.
Features that are related to diagnoses and calculated jointly on Current Procedural Terminology (CPT) and Healthcare Common Procedure Coding System (HCPCS) procedure codes along with ICD-9 and ICD-10 diagnosis codes	Cataract; and sinusitis.

Features related to the problem list	*No. of active problems; no. of active problems of wheezing; no. of active problems of asthma with (acute) exacerbations; no. of active problems of asthma; no. of active problems of obesity; no. of active problems of congestive heart failure; no. of active problems of sleep apnea; no. of active problems of hypertension; no. of active problems of chronic obstructive pulmonary disease; no. of active problems of rhinitis; no. of active problems of diabetes; no. of active problems of anxiety/depression; no.of active problems of gastroesophageal reflux disease; no. of active problems about smoking; and the priority of the last active problem of asthma*.
Features related to medications	Total no. of medications ordered; no. of medication orders; total no. of differing medications ordered; total no. of medication refills permitted; total no. of units of medications ordered; total no. of asthma medications ordered; no. of asthma medication orders; total no. of differing asthma medications ordered; total no. of asthma medication refills permitted; total no. of units of asthma medications ordered; total no. of short-acting beta-2 agonists ordered; total no. of refills permitted for short-acting beta-2 agonists; total no. of units of short-acting beta-2 agonists ordered; total no. of systemic corticosteroids ordered; total no. of refills permitted for systemic corticosteroids; total no. of units of systemic corticosteroids ordered; *no. of asthma reliever orders; total no. of asthma relievers ordered; total no. of refills permitted for asthma relievers; total no. of differing asthma relievers ordered; total no. of units of asthma relievers ordered*; total no. of units of asthma relievers ordered that are neither short-acting beta-2 agonists nor systemic corticosteroids; total no. of asthma relievers ordered that are neither short-acting beta-2 agonists nor systemic corticosteroids; *no. of asthma controller orders; total no. of asthma controllers ordered; total no. of units of asthma controllers ordered; total no. of differing asthma controllers ordered; total no. of refills permitted for asthma controllers*; total no. of inhaled corticosteroids ordered; total no. of refills permitted for inhaled corticosteroids; total no. of units of inhaled corticosteroids ordered; total no. of mast cell stabilizers ordered; total no. of refills permitted for mast cell stabilizers; total no. of units of mast cell stabilizers ordered; whether nebulizer was used; *total no. of nebulizer medications ordered; no. of nebulizer medication orders; total no. of units of nebulizer medications ordered; total no. of differing nebulizer medications ordered; total no. of refills permitted for nebulizer medications*; and whether spacer was used.
Features related to insurances	Whether the patient had any public insurance on the last day; whether the patient had any private insurance on the last day; and whether the patient was paid by oneself or a charity on the last day.
Features related to the patient's visit types	No. of ED visits; *no. of ED visits related to asthma*; the most recent ED visit's length of stay; the mean length of stay of an ED visit; no. of visits; no. of outpatient visits to the patient's PCP; no. of outpatient visits; no. of outpatient visits whose primary diagnosis is asthma; no. of hospitalizations; the hospitalizations' total length; the mean length of a hospitalization; no. of hospitalizations, ED visits, and outpatient visits; no. of intensive care admissions; the most emergent admission type of all of the visits; the most recent visit's admission type (elective, urgent, emergency, or trauma); no. of major visits for asthma; and *the most recent visit's type (ED visit, outpatient visit, or hospitalization)*. As in our prior paper [23], we defined a major visit for asthma as an ED visit having an asthma diagnosis code, a hospitalization having an asthma diagnosis code, or an outpatient visit having a primary diagnosis of asthma. An outpatient visit having only a secondary diagnosis of asthma was treated as a minor visit for asthma.
Features on visit status and appointment scheduling	The day of the week when the most recent ED visit began; no. of cancelled appointments; the most recent visit's discharge disposition location (home, left against medical advice, or other non-home location); no. of no shows; no. of times of leaving against medical advice; for the most recent visit, the time to the actual visit after making the request showing its urgency; across all of the visits, the shortest time to the actual visit after making the request; whether the most recent hospitalization came from the ED; no. of days from the most recent hospitalization; *no. of visits having same day appointments*; no. of days from the most recent ED visit; no. of days from the most recent outpatient visit; *no. of days from the most recent ED visit on asthma; and no. of days from the most recent outpatient visit on asthma*.
Features describing the patient's care continuity degree	No. of differing asthma medication prescribers; no. of differing EDs the patient went to; no. of differing medication prescribers; no. of differing providers the patient saw in outpatient visits; and no. of differing PCPs of the patient.

Features related to procedures	Mechanical ventilation reflected by ICD-10 and ICD-9 procedure codes; no. of ICD-10 and ICD-9 procedure codes; no. of HCPCS procedure codes of home oxygen therapy; no. of CPT procedure codes of the fractional exhaled nitric oxide test; no. of CPT/HCPCS procedure codes; no. of CPT procedure codes of pulmonary function tests; and no. of CPT/HCPCS procedure codes of influenza vaccination.
Allergy features	Indicator of drug or material allergy; *the greatest severity of the patient's drug or material allergies*; indicator of environmental allergy; *the greatest severity of the patient's environmental allergies*; indicator of food allergy; *the greatest severity of the patient's food allergies*; and no. of the patient's allergies.
Features related to pulmonary function tests	*The mean forced expiratory volume in 1 second (FEV1); and the lowest FEV1.*
Features related to social behavior history	*No. of fluid ounces of alcohol the patient consumed every week according to the most recent record; whether the patient was ever documented of consuming alcohol; whether the patient consumed alcohol according to the most recent record; the mean no. of fluid ounces of alcohol the patient consumed every week across all of the records; no. of alcohol drinks the patient consumed every week according to the most recent record; the mean no. of alcohol drinks the patient consumed every week across all of the records; no. of packs of cigarettes the patient consumed every day according to the most recent record; whether the patient was a smoker according to the most recent record; the mean no. of packs of cigarettes the patient consumed every day across all of the records; whether the patient was a former smoker according to the most recent record; no. of years the patient had smoked for according to the most recent record; no. of times the patient took illicit drugs every week according to the most recent record; whether the patient took any illicit drug according to the most recent record; the mean no. of times the patient took illicit drugs every week across all of the records; and whether the patient was ever documented of taking any illicit drug.*
Provider features	We defined the patient's PCP known at the most recent clinic visit as the patient's current PCP. We considered the following PCP features: *no. of years that the PCP had practiced at UWM for*; the PCP's age; whether the patient is of the same gender as the PCP; the PCP's primary specialty; whether the PCP is a resident; the PCP's type (physician, nurse, physician assistant, or other); the PCP's clinician title (doctor of medicine, registered nurse, physician assistant, or other); the percentage of the PCP's asthmatic patients in the pre-index year incurring asthma hospital encounters in the index year; and no. of asthmatic patients of the PCP.

Table 2. The features used in our final UWM predictive model and their importance values.

Rank	Feature	Importance calculated as the feature's apportioned contribution to the model
1	No. of ED visits related to asthma	0.2113
2	The mean length of stay of an ED visit	0.1088
3	No. of days from the most recent ED visit	0.1075
4	No. of primary or principal asthma diagnoses	0.0986
5	No. of days from the most recent diagnosis of asthma with (acute) exacerbation or status asthmaticus	0.0798
6	Whether the patient is black	0.0423
7	No. of ED visits	0.0368
8	No. of asthma diagnoses	0.0322
9	No. of years from the first encounter related to asthma in the data set	0.0233
10	No. of nebulizer medication orders	0.0174
11	Whether the patient is white	0.0148
12	The highest systolic blood pressure	0.0128
13	No. of CPT/HCPCS procedure codes	0.0120
14	The largest BMI	0.0107
15	The most recent ED visit's length of stay	0.0104
16	Whether the patient is married	0.0102

17	Total no. of units of medications ordered	0.0101
18	No. of asthma medication orders	0.0087
19	Whether nebulizer was used	0.0082
20	No. of no shows	0.0081
21	Total no. of differing asthma medications ordered	0.0078
22	The mean heart rate	0.0072
23	No. of diagnoses of asthma with (acute) exacerbation	0.0070
24	Whether the patient had any private insurance on the last day	0.0060
25	The mean respiratory rate	0.0058
26	No. of days from the most recent asthma diagnosis	0.0056
27	For the most recent visit, the time to the actual visit after making the request	0.0054
28	The mean systolic blood pressure	0.0053
29	Total no. of differing medications ordered	0.0046
30	Whether the patient has any drug or material allergy	0.0046
31	Whether the patient had any public insurance on the last day	0.0042
32	The lowest SpO_2	0.0040
33	No. of active problems	0.0038
34	Whether the most recent visit is an ED visit	0.0038
35	The highest temperature	0.0037
36	No. of laboratory tests	0.0034
37	No. of asthma controller orders	0.0029
38	No. of visits	0.0029
39	Total no. of short-acting beta-2 agonists ordered	0.0029
40	The largest blood eosinophil count	0.0028
41	No. of asthma medication orders	0.0027
42	Total no. of asthma relievers ordered that are neither short-acting beta-2 agonists nor systemic corticosteroids	0.0025
43	The mean temperature	0.0022
44	Total no. of inhaled corticosteroids ordered	0.0020
45	No. of days from the most recent outpatient visit on asthma	0.0020
46	The severity of the most recent asthma diagnosis	0.0020
47	Total no. of refills permitted for short-acting beta-2 agonists	0.0019
48	No. of differing providers the patient saw in outpatient visits	0.0019
49	Age	0.0019
50	No. of outpatient visits to the patient's PCP	0.0019
51	No. of laboratory tests having abnormal results	0.0018
52	Total no. of systemic corticosteroids ordered	0.0016
53	Whether the most recent asthma diagnosis is a primary or principal one	0.0014
54	Whether the patient is single	0.0013
55	The day of the week when the most recent ED visit began	0.0013
56	The relative change of BMI	0.0013
57	The mean length of a hospitalization	0.0012
58	No. of days from the most recent ED visit on asthma	0.0011
59	No. of active problems of asthma	0.0010
60	Total no. of differing nebulizer medications ordered	0.0010
61	Total no. of differing asthma relievers ordered	0.0010
62	Total no. of refills permitted for asthma controllers	0.0010
63	Whether the patient has any mental disorder	0.0009
64	The relative change of weight	0.0008
65	Whether the most recent visit's admission type is emergency	0.0007
66	Whether the patient was a smoker according to the most recent record	0.0007
67	Whether the most recent hospitalization came from the ED	0.0006
68	The severity of the most severe asthma diagnosis	0.0005
69	No. of outpatient visits	0.0005

70	Total no. of medication refills permitted	0.0005
71	The highest diastolic blood pressure	0.0005

Abbreviations:

BMI: body mass index
CPT: Current Procedural Terminology
FEV1: forced expiratory volume in 1 second
FVC: forced vital capacity
HCPCS: Healthcare Common Procedure Coding System
IgE: immunoglobulin E
SpO_2: peripheral capillary oxygen saturation
